# HIV, Stigma, and Rates of Infection: A Rumour without Evidence: Authors' Reply

**DOI:** 10.1371/journal.pmed.0040044

**Published:** 2007-01-30

**Authors:** Daniel D Reidpath, Kit Yee Chan

The HIV/AIDS area has always been highly politically and emotionally charged, and we wrote a controversial and provocative piece. Most of the responses to it were unreasoned. The most cogent response came from UNAIDS (the Joint United Nations Programme on HIV/AIDS) [1], and it generally restated an already well articulated position. We disagree with a number of the points for the reasons discussed in our original essay, and applaud one point.

First, a brief restatement of our argument is warranted. There is good evidence that HIV-related stigma adversely affects the lives of people living with HIV/AIDS. There is little or no evidence, however, to support the notion that HIV-related stigma is one of the determinants of the global HIV epidemic. Furthermore, an argument could be made for why stigma might slow or contain the spread of infection in the general population. Given the very different effect the two positions would have on policy and the significance of the HIV epidemic, they deserve investigation. Among epidemiologists, two competing hypotheses, for which there is no strong evidence either way, would constitute a position of equipoise.

The UNAIDS position is that HIV stigma and discrimination is a human rights violation “and should be stopped for that reason alone” [1]. Excellent point! Let's do that and understand why we are doing it. But in the absence of evidence, do not let us conflate the epidemiology of the infection with the human rights position. The letter goes on to say that “UNAIDS cannot endorse a hypothesis that….” Of course, we never wanted a hypothesis endorsed. We want hypotheses tested. The original (UNAIDS) position is treated as fact, and from the UNAIDS response, continues to be treated as a fact, when it was (and is) simply a hypothesis. Before this or any other hypothesis is endorsed, it should be tested.

The role of stigma is complex, carrying with it social benefits and social harms. It is a social process. By treating it as a serious object of health research, its multiple roles, including its role in disease propagation, can be legitimately investigated rather than marginalized as the poster child of advocates. Furthermore, by understanding the nature of stigma, it may be possible to develop health interventions that neither rely on stigma to succeed nor arbitrarily and inappropriately declare it to be a causal agent.

Research and advocacy have important and fundamental roles in population health. They each need to be used appropriately.
